# Microbiome composition and turnover in the face of complex lifecycles and bottlenecks: insights through the study of dung beetles

**DOI:** 10.1128/aem.01278-24

**Published:** 2024-12-20

**Authors:** Joshua A. Jones, Irene Garcia Newton, Armin P. Moczek

**Affiliations:** 1 Department of Biology, Indiana University Bloomington 123993 , Bloomington, Indiana, USA; University of Illinois Urbana-Champaign, Urbana, Illinois, USA

**Keywords:** microbiota, transmission, assembly, development, dung beetles

## Abstract

Microbiome composition and function often change throughout a host’s life cycle, reflecting shifts in the ecological niche of the host. The mechanisms that establish these relationships are therefore important dimensions of host ecology and evolution; yet, their nature remains poorly understood. Here, we sought to investigate the microbial communities associated with the complex life cycle of the dung beetle *Onthophagus taurus* and the relative contributions of host life stage, sex, and environment in determining microbiome assembly. We find that *O. taurus* plays host to a diverse microbiota that undergo drastic community shifts throughout host development, influenced by host life stage, environmental microbiota, and, to a lesser degree, sex. Contrary to predictions, we found that egg and pupal stages—despite the absence of a digestive tract or defined microbe-storing organs—do not constrain microbial maintenance, while host-constructed environments, such as a maternally derived fecal pellet or the pupal chamber constructed by late larvae, may still serve as complementary microbial refugia for select taxa. Lastly, we identify a small community of putative core microbiota likely to shape host development and fitness. Our results provide important insights into mechanisms employed by solitary organisms to assemble, maintain, and adjust beneficial microbiota to confront life-stage-specific needs and challenges.

**IMPORTANCE:**

As the influence of symbionts on host ecology, evolution, and development has become more apparent so has the importance of understanding how hosts facilitate the reliable maintenance of their interactions with these symbionts. A growing body of work has thus begun to identify diverse behaviors and physiological mechanisms underpinning the selective colonization of beneficial symbionts across a range of host taxa. Yet, how organisms with complex life cycles, such as holometabolous insects, establish and maintain key symbionts remains poorly understood. This is particularly interesting considering the drastic transformations of both internal and external host morphology, and the ecological niche shifts in diet and environment, that are the hallmark of metamorphosis. This work investigates the dynamic changes of the microbiota associated with the complex life cycle and host-constructed environments of the bull-headed dung beetle, *Onthophagus taurus*, a useful model for understanding how organisms may maintain and modulate their microbiota across development.

## INTRODUCTION

Multicellular organisms frequently host diverse communities of beneficial microbial organisms able to positively influence host fitness across a range of contexts, such as resource utilization, developmental signaling, and defense (1–4) In some instances, such benefits can be obtained from the general presence of microbes. For example, colonization resistance against pathogens in the mammalian gut and hypoxia-induced molting in mosquitoes can both be conferred by a range of nonspecific microbial communities (5, 6). In others, however, hosts have evolved cooperative relationships with specific members of the microbial community. Examples include *Bifidobacterium*-mediated priming of the infant immune system (7), *Buchnera* synthesizing amino acids needed to support aphid development (8), or *Rhizobium* fixing nitrogen in legumes (9). These specific interactions, in turn, necessitate the evolution of mechanisms that facilitate the reliable transmission of associations between hosts and symbionts across generations (4, 10). Common mechanisms include (i) vertical transmission, as is seen in aphids and burying beetles, which transmit symbionts directly or indirectly from parent to offspring (8, 11, 12); (ii) horizontal transmission, such as in legumes and bobtail squids, which selectively update their respective symbionts from the microbial communities in their environments (13–16); or (iii) some combination of the two, as in humans which receive symbionts from both parents and their environment (17). The mechanisms that establish and maintain host-symbiont relationships are therefore important dimensions of host ecology and evolution yet remain poorly understood outside of a few model organisms.

Establishing and maintaining host-symbiont relationships is further shaped by the complexity of the host’s life cycle. For instance, holometabolous insects transition through distinct life stages (egg, larva, pupa, and adult), often involving the radical transformation of both internal and external host morphology (18). In addition, holometabolous metamorphosis is commonly paralleled by drastic ecological niche shifts including diet (e.g., mosquitoes feed on detritus as larvae, followed by blood and nectar as adults [19]), environmental conditions (e.g., the subterranean larvae and pupae of beewolves and dung beetles both of which transform into free-living adults [20, 21]), and the nature of interactions with conspecifics (e.g., solitary larvae that metamorphose into social adults [22]). A growing body of work suggests that these ontogenetic niche shifts often coincide with shifts in the associated microbiota and resulting host-microbe interactions (4, 10), creating opportunities to tailor microbiota composition to suit the life-stage-specific needs of the host. However, whether these shifts in microbial community composition are under host control, shaped by symbiont behavior, or simply a byproduct of changing ontogenetic environments remains largely unknown. In particular, the role of transitional stages (such as eggs and pupae) in shaping host stage-specific transitions in microbiome assembly remains to be explored. This may be of particular importance as these stages typically lack defined digestive systems in which to store and maintain symbionts (though research on pupal organ structures is limited [23, 24]) and may be especially susceptible to infection from pathogens ([25, 26]). The inability to internally maintain symbionts may therefore cause these stages to act as bottlenecks to the maintenance of symbionts across the life cycle (4). Similarly, a host’s level of sociality (solitary, gregarious, or (eu)social) may shape mechanisms available to maintain symbionts throughout development. Whereas gregarious or social insects may acquire select microbiota from conspecifics with ease (27, 28), this route of transmission is not available to solitary species thus necessitating alternative mechanisms to ensure the reliable passage of symbionts across generations and throughout development for the vast majority of holometabolous insects. Here we utilize the complex, holometabolous life cycle of onthophagine dung beetles to begin exploring mechanisms shaping the assembly and composition of life-stage-specific microbial communities.

**Fig 1:**
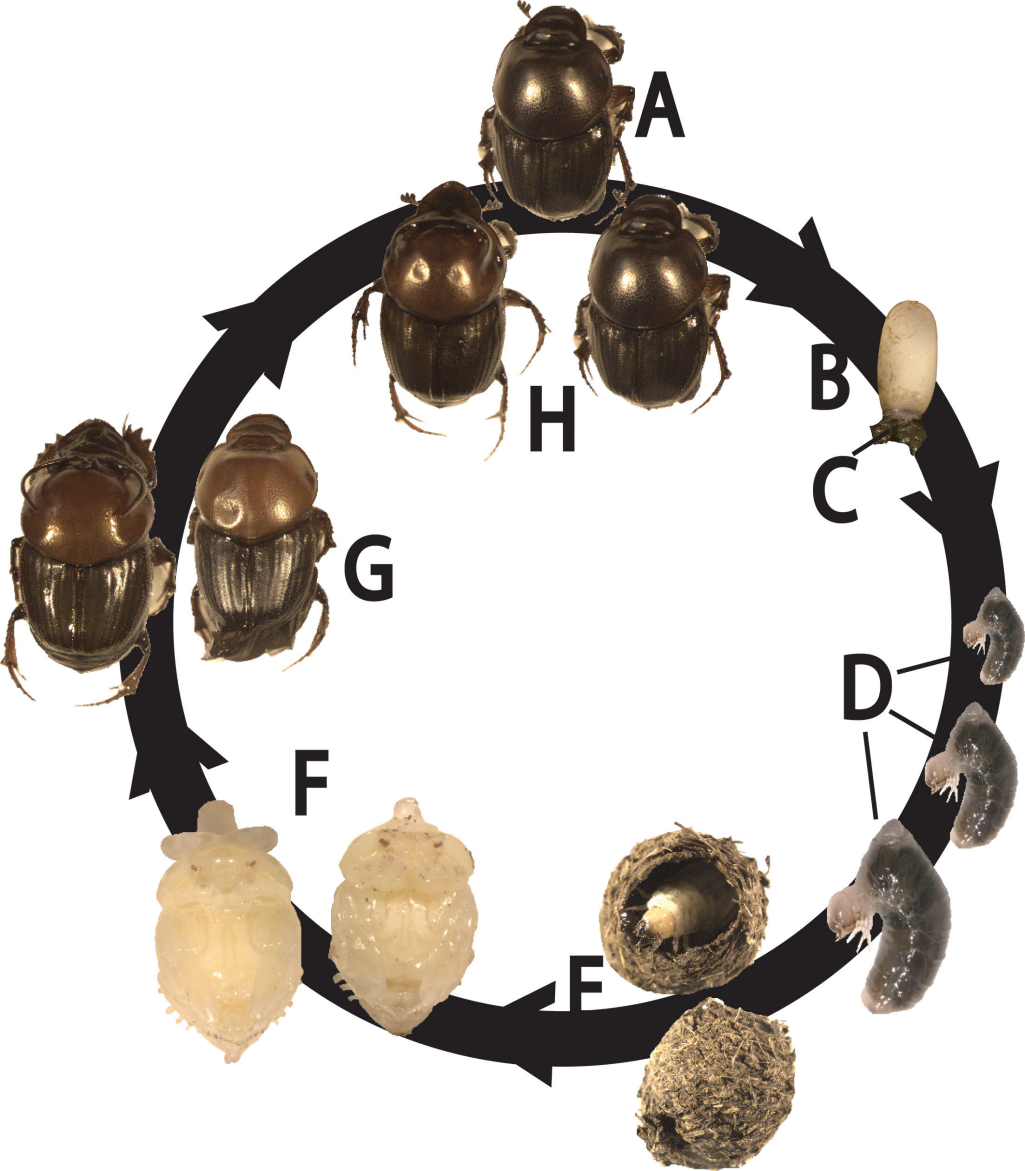
Host and host-associated samples collected throughout the *O. taurus* life cycle: Mature adult female beetles (A: mothers) that construct subterranean brood balls in which they oviposit a single egg (**B**). Eggs rest atop (**C**) pedestals, that is, fecal pellets that mothers construct and deposit prior to oviposition and that (**D**) larvae consume prior to feeding on the brood ball. Larvae progress through three larval instars while continually feeding within the brood ball, defecating into the brood ball, and re-consuming the mixture (21). At the end of their final instar larvae purge their guts and use the contents to construct a (**E**) pupal chamber in which they enter their (**F**) pupal stage. After eclosing as an adult (G: young adults) the beetle remains within the brood ball until their cuticle hardens, possibly feeding on the pupal chamber and remaining dung. Once the cuticle has hardened the beetle emerges and seeks dung pads to congregate with other beetles, feed, and reproduce (H: mature adults), continuing the cycle.

*Onthophagus* dung beetles spend the entirety of their development (Fig. 1) in complete isolation from conspecifics. Specifically, adult females bury dung underground to form so-called brood balls, into which they deposit a single egg (21). Larvae hatch into this environment and begin to consume the brood ball while continually adding and spreading their feces, then re-feeding on the increasingly modified composite, all while in complete isolation from conspecifics (21). Within the brood ball, larvae progress through three larval instars before purging their gut content from which they form a pupal chamber and within which they pupate. Upon hatching as an adult, the beetle remains in the brood ball until its cuticle hardens, possibly feeding on the pupal chamber and remaining dung. Once cuticle hardening is complete the beetle will emerge and seek dung pads to congregate, feed, and reproduce.

Most *Onthophagus* beetles are obligately reliant on herbivore dung for both food and reproduction. While larvae consume fibrous, cellulose-rich components of the dung, adult beetles feed on nitrogen-rich, liquid portions. Herbivore dung is a diet poor in essential nutrients and comprised primarily of tough-to-digest carbohydrates such as cellulose and xylan (29–31). As with many other insects reliant on complex and nutrient-poor diets, *Onthophagus* beetles are likely dependent on microbial symbionts to properly derive nutrients from their diet. In *Onthophagus*, the relevant microbiota are passed vertically from mother to offspring *via* a fecal secretion (known as the *pedestal*) which is deposited prior to oviposition and consumed by the larvae after hatching (21). Depriving offspring of the pedestal slows larval growth and decreases survival and adult size, effects which are amplified under stress yet rescued by inoculation with bacteria isolated from the pedestal (32). Hosts experience developmental deficits when receiving soil bacteria (32), bacteria from distantly related dung beetles (33), as well as, to a lesser degree, closely related species (34). Past work thus suggests that *Onthophagus* species have evolved a certain degree of specificity in their reliance on microbial associations; yet, how these associations are established and maintained in the face of their complex life cycle and possible life stage bottlenecks is unknown.

Here, we sought to investigate the microbial communities associated with the *O. taurus* life cycle and the relative contribution of the environment experienced and, in part, created by the developing host itself in determining microbiome assembly. We also determine what role properties of the host, such as life stage and sex, play in determining microbiota composition as well as which microbial taxa may represent core members of the community. Lastly, we tested whether—on one side—the egg and pupal stages might act as bottlenecks and therefore constrain microbiome transmission, and—on the other—whether the maternally derived pedestal and the late larva-derived pupal chamber may serve as microbiome reservoirs able to facilitate microbial transmission across these stages.

## RESULTS

We sequenced 82 samples across six life stages, two organism-derived structures (summarized in Fig. 1), as well as the dung given to mothers for reproduction and the dung used in the 12-well plates in which the offspring beetles developed. Sequencing of these samples resulted in a total of 554,906 clusters. Reads per sample ranged from 51 to 19,558 and after assembly and quality control steps all samples were rarefied to 1,013. This rarefaction maintained enough samples for later analyses, yet, as rarefaction curves did not flatten (Fig. S1), probably obscured rare taxa that would be observed at greater sequencing depths. Rarefied samples yielded 2,795 OTUs (operational taxonomic units) at 97% similarity, 346 genera, 155 families, 64 orders, 34 classes, and 17 phyla. Final sample sizes are listed in Table S1 and data files listing OTU abundances across samples, and OTU classifications, are available in our Data Set S2).

### Bacterial composition changes cyclically throughout development

**Fig 2:**
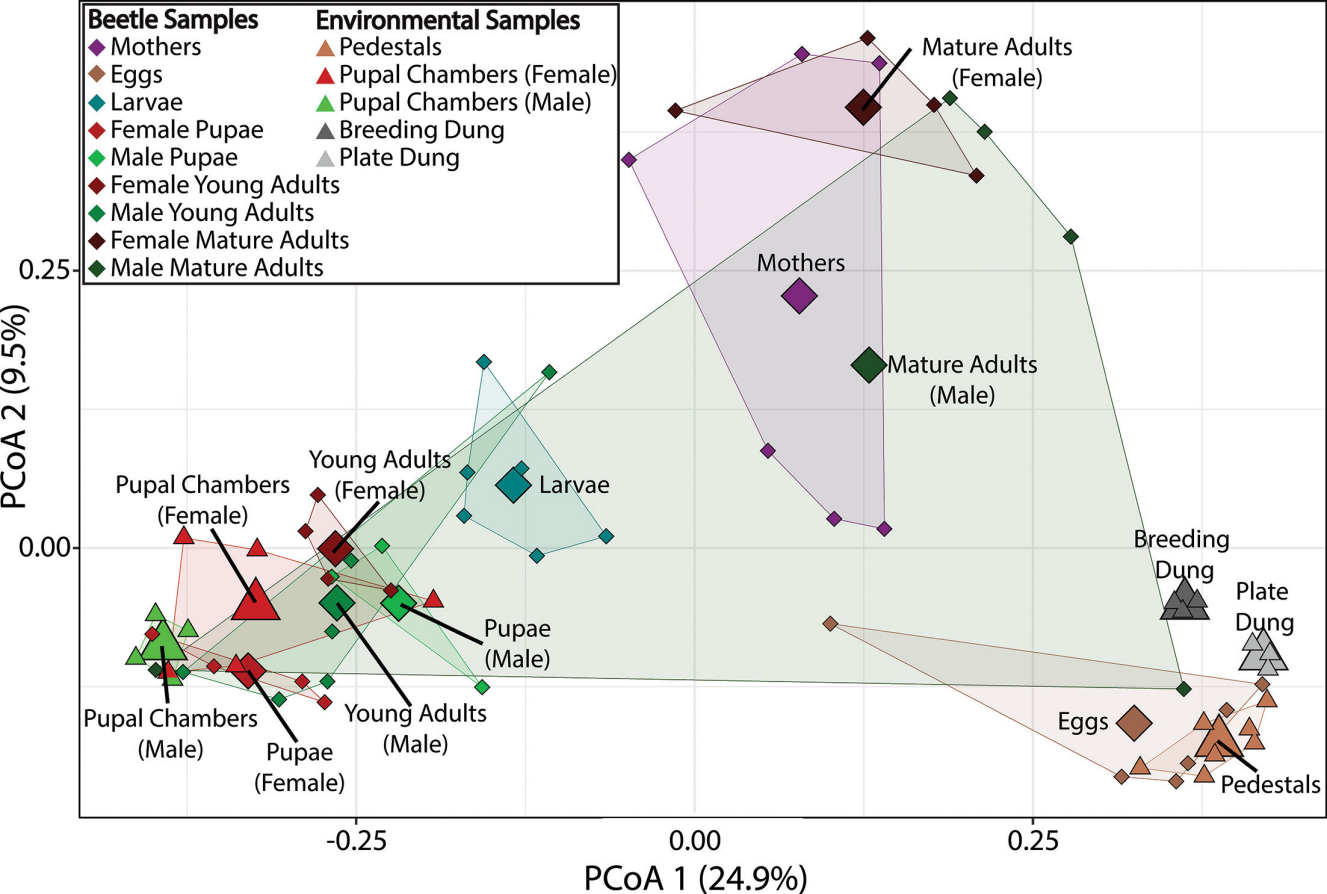
Differentiation of microbial communities harbored within beetle life stages and environments. Shown are Bray-Curtis Dissimilarites between samples across axes 1 and 2 of a principal coordinate analysis. A PERMANOVA reveals that life stage has a significant effect on microbiome composition (Pseudo-F = 4.44, R^2^ = 0.457, *P* = 0.001) as does sample type generally (Bray-Curtis: Pseudo-F = 4.74, R^2^ = 0.542, *P* = 0.001). Sample types are differentiated by colors, diamonds represent beetle samples, triangles represent environmental samples, circles represent each sample type’s centroid, and colored lines depict a hull outlining sample types.

We sought to test whether dung beetle life stages significantly affected the composition of bacterial communities at the OTU level. A PERMANOVA test including all beetle samples showed that life stage has a significant effect on both the bacterial taxa associated with a given beetle life stage (Jaccard) and their relative abundance (Bray-Curtis) (Jaccard: Pseudo-F = 2.54, R^2^ = 0.367, *P* = 0.001, Bray-Curtis: Pseudo-F = 4.44, R^2^ = 0.457, *P* = 0.001, Fig. 2; Fig. S2 to 4). When other samples are included (pedestals, pupal chambers, breeding, and plate dung), we continue to see a characteristic microbiome-by-sample difference (PERMANOVA; Jaccard: Pseudo-F = 3.18, R^2^ = 0.443, *P* = 0.001, Bray-Curtis: Pseudo-F = 4.74, R^2^ = 0.542, *P* = 0.001). Next, we conducted stage-to-stage comparisons across beetle samples and found significant differences (Bray-Curtis dissimilarity; Data Set S2) between all life stage transitions with only two exceptions: the microbial community associated with male pupae did not differ from that of young male adults (Pseudo-F = 1.15, R^2^ = 0.14117, *P* = 0.341), while the microbial community associated with mothers, whose offspring were used to generate the remaining life stages, did not differ significantly from that of mature adult females or mature adult males generated at the end of the experiment (mothers—mature adult females: Pseudo-F = 1.87, R^2^ = 0.1892, *P* = 0.061; mothers—mature adult males: Pseudo-F = 1.50, R^2^ = 0.14247, *P* = 0.133). As such, life-stage-specific microbial communities appear to undergo a cyclical change throughout development, diverging across subsequent life stages while converging back to that of maternal females in adulthood.

### Sexes diverge in bacterial composition only as young adults

We tested the hypothesis that male and female adults may diverge in microbiome composition by testing for differences in bacterial composition and dispersion of those life stages where sex can be ascertained unambiguously (i.e., pupae, young adults, and mature adults). There was a significant difference between sexes in Bray-Curtis dissimilarity in young adults (Pseudo-F = 1.94, R^2^ = 0.19486, *P* = 0.023) yet there was no significant effect in pupae (Pseudo-F = 1.08, R^2^ = 0.17742, *P* = 0.423) or mature adults (Pseudo-F = 1.08, R^2^ = 0.13326, *P* = 0.369). A beta dispersion test revealed no significant difference in dispersion between sexes in Bray-Curtis dissimilarity (BetDisper; Pupae: F = 0.0078, *P* = 1.0000; Young Adult: F = 0.36387, *P* = 0.2769; Mature Adult: F = 1.3854, *P* = 0.8391) or Jaccard similarity (BetDisper; Pupae: F = 0.2389, *P* = 1.000; Young Adult: F = 4.4985, *P* = 0.1797; Mature Adult: F = 1.5054, *P* = 0.7131) suggesting that the host-associated bacterial communities of males are no more variable than those of females of the same stage.

### Life stages differ in the degree to which their microbiota is influenced by environmental microbes

**Fig 3:**
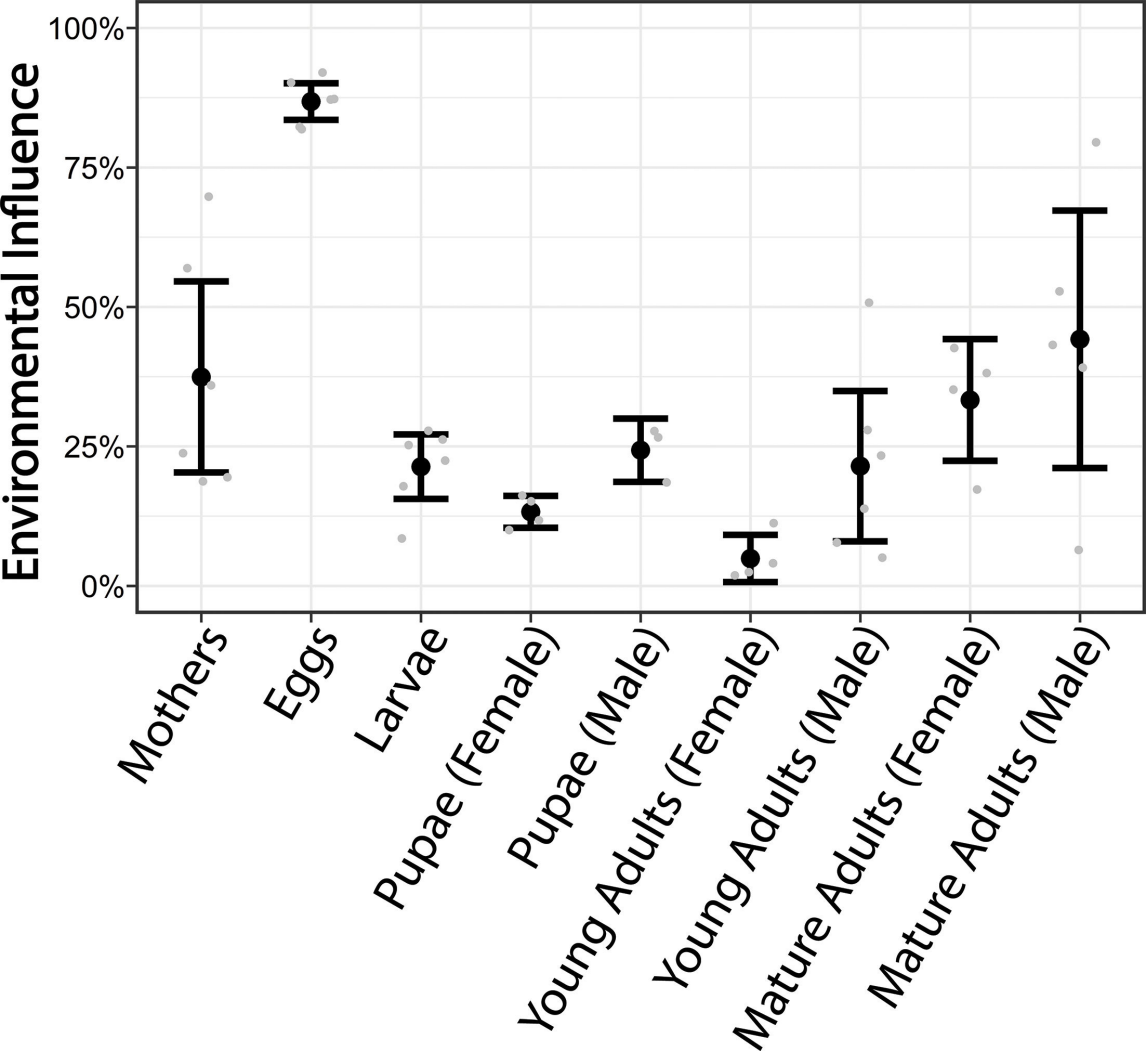
Influence of environmental bacteria on bacterial communities across *O. taurus* life stages. Life stages significantly differ in how much of their bacterial community is composed of environmental bacteria (F = 14.693, *P* < 0.001) which is largely driven by the egg stage. Shown are means and 95% confidence intervals of the proportions of the total relative abundance of bacterial taxa within each sample present in dung samples (breeding and plate dung samples). Gray points represent individual samples.

To estimate the role of environmental microbiota in microbiome assembly, we determined the compositional similarities between bacterial communities associated with each life stage and that of the dung prior to contact with the beetle. Each life stage harbored bacterial communities significantly different from those contained within the dung, with the exception of male pupae (Pseudo-F = 7.10, R^2^ = 0.63967, *P* = 0.1) and male mature adults (Pseudo-F = 2.74, R^2^ = 0.31374, *P* = 0.063). To further assess the influence of environmental sources on microbiome composition, each OTU was divided into two categories based on its presence/absence across dung samples. Those bacteria present in at least one dung sample were considered “environmental” while those not found in dung samples were considered “beetle-associated.” Life stage significantly predicted how much of the bacterial community was comprised of environmental bacteria (ANOVA: F = 14.693, *P* > 0.001, Fig. 3). However, this effect was mostly driven by the egg stage, whose microbial community was significantly more enriched with environmental bacteria than other life stages (TukeyHSD: *P* < 0.001), and to a lesser degree by mothers possessing a significantly higher proportion of environmental bacteria than young adult females (TukeyHSD: *P* = 0.0322). Overall, this suggests that diet-derived microbes can influence the host microbiome but that the magnitude of this influence changes across development.

### Several OTUs found across each life stage may reflect putative core bacteria

To determine whether *O. taurus* possesses a core microbiome retained across all life stages, we identified which bacterial OTUs were found in at least one individual from each life stage. These were further differentiated into beetle-associated, those found across life stages but not in the dung, and environmental, those found across life stages and in the dung. Beetle-associated OTUs included one OTU each of *Brucella* (*Brucellaceae*), *Variovorax* (Comamonadaceae), *Chryseobacterium* (*Weeksellaceae*), *Paracoccus* (*Rhodobacteraceae*), and an unclassified Microbacteriaceae, as well as two *Dysgonomonas* (*Dysgonomonadaceae*) OTUs (Fig. S5). Environmental core OTUs included one OTU each of *Clostridium* (*Lachnospiraceae*), *Sphingobacterium* (*Sphingobacteriaceae*), *Nocardiodides* (*Nocardioidaceae*), and *Turicibacter* (*Turicibacteraceae*), one OTU each of unclassified Comamonadaceae, and Microbacteriaceae, as well as two OTUs each of *Planococcaceae* and *Acinetobacter* (*Moraxellaceae*) (Fig. S6). Lastly, we conducted an indicator species analysis to identify which bacterial genera are indicative of a sample type or groups of sample types. A cumulative list of OTUs and genera, the groups they indicate, and the relevant test statistics can be found in Data Set S2. These results identify several members of a putative *O. taurus* core microbiome and the developmental time points when they are most consistently detected.

### Three bacterial phylotypes dominate the beetle life cycle

**Fig 4:**
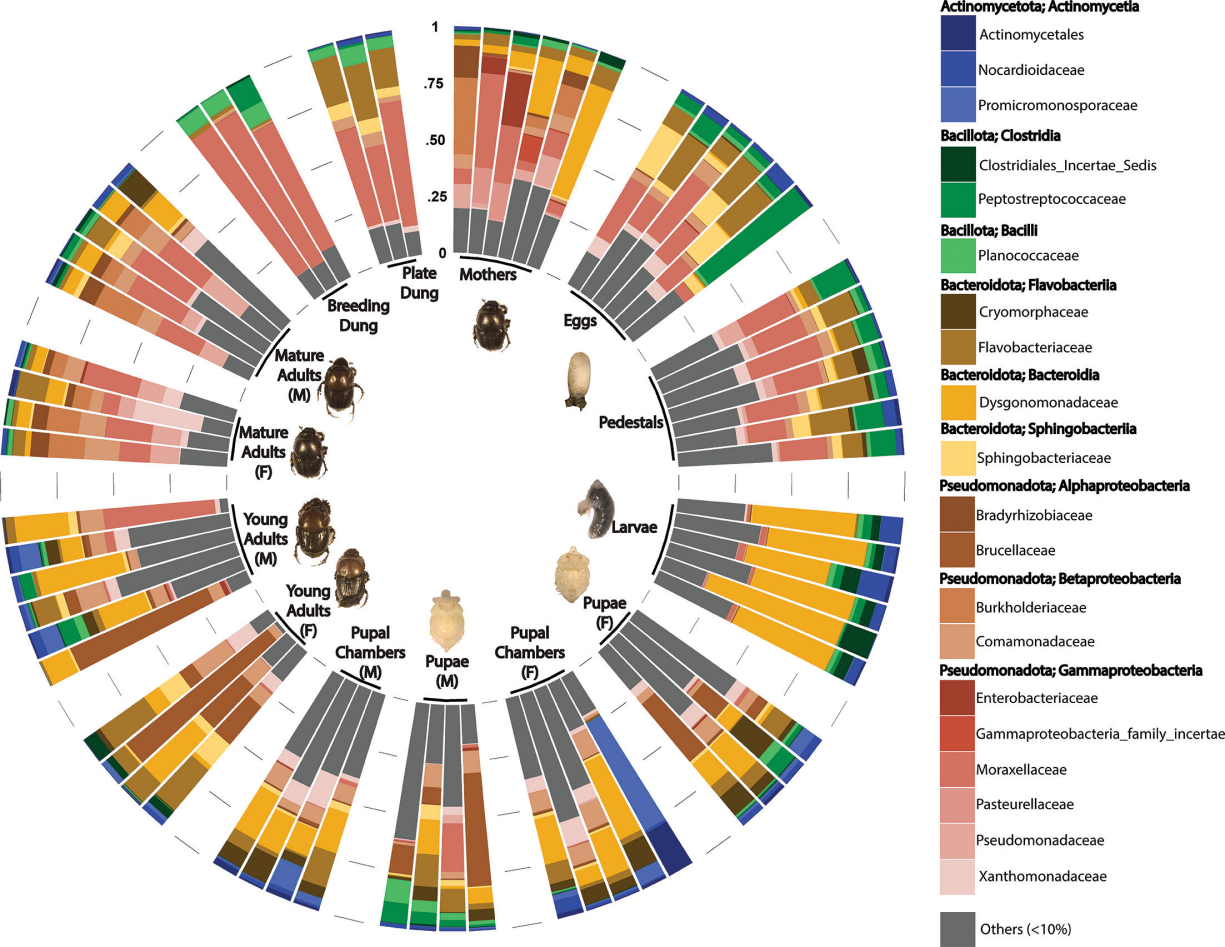
Relative abundance of bacterial families across each sample. Samples are grouped based on sample type and shown clockwise in order of life stages and associated host-constructed environments. Microbial families are differentiated by color and depict those that represent at least 10% of reads from any sample.

**Fig 5:**
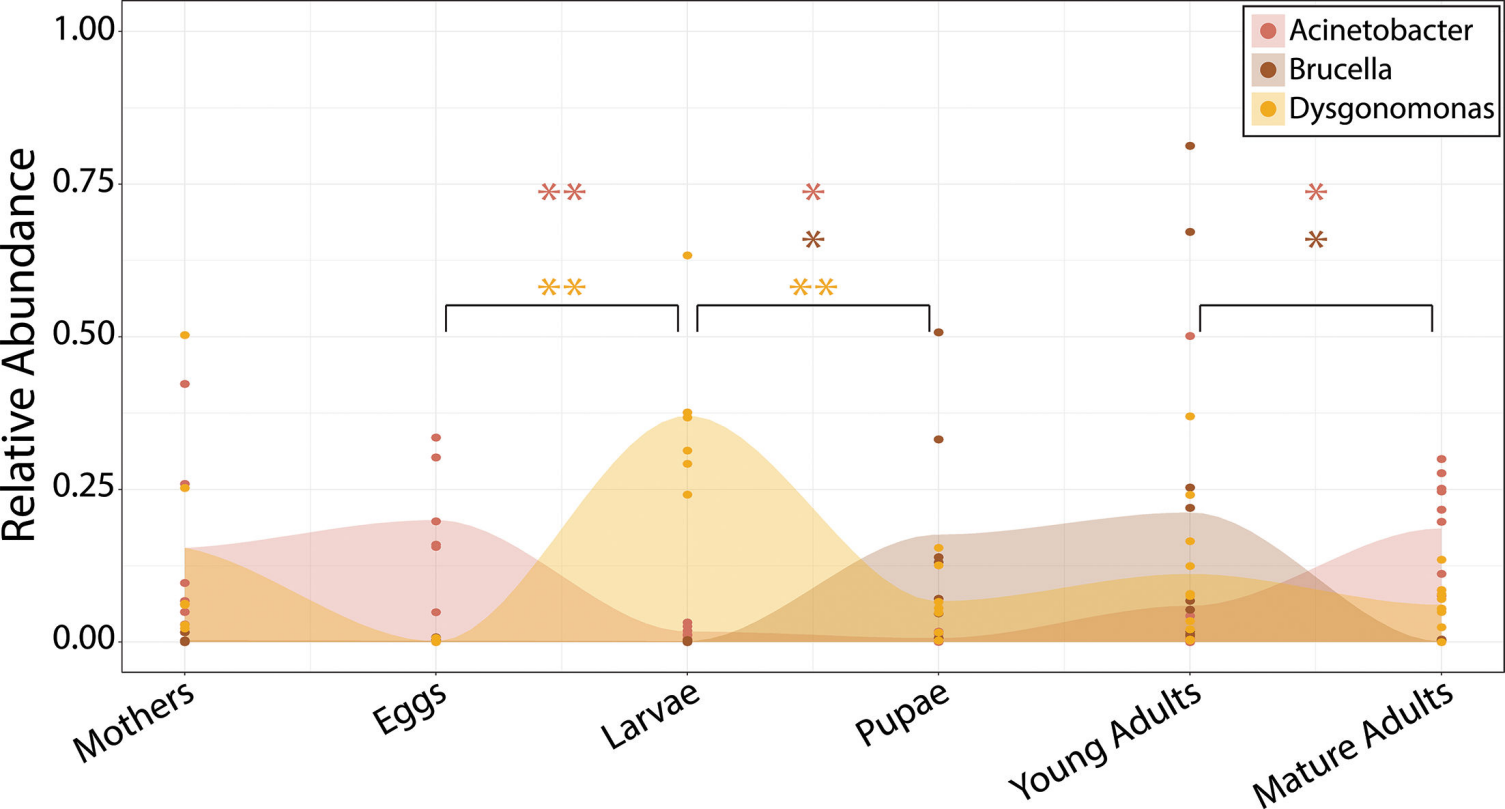
Dominant bacteria across the *O. taurus* life cycle. *Acinetobacter*, *Brucella*, and *Dysgonomonas* constitute the most abundant microbial genera across the *O. taurus* life stages. Colors represent microbial taxa, points represent relative abundances within individual samples, and asterisks denote significant changes in average relative abundance across subsequent life stages (Welch’s *t*-test: *P ≤ 0.05, **P ≤ 0.01).

To further our understanding of the community members driving stage divergences, and to highlight potentially beneficial members of the microbiome, we identified bacterial taxa that dominate each life stage’s community. Throughout all samples, 20 bacterial families appeared in high abundance (>10%) in at least one sample (Fig. 4). These families represent four phyla, nine classes, and twenty-six genera (Fig. S7). In particular, *Acinetobacter, Brucella,* and/or *Dysgonomonas* emerged as the most dominant genera within any sampled life stage (Fig. 5). Specifically, *Acinetobacter* were the most abundant in eggs and mature adults while *Dysgonomonas* were most abundant in larvae. The remaining life stages were dominated by combinations of bacteria with mothers being dominated by *Acinetobacter* and *Dysgonomonas,* whereas pupae and young adults harbored communities composed mainly of both *Brucella* and *Dysgonomonas*. It is important to note that in this analysis, *Dysgonomonas* comprises a diverse community of 208 OTUs, in contrast to the simpler communities of *Acinetobacter* (54 OTUs), and Brucella (one single OTU, though we detected at least eight different OTUs of unclassified *Brucellaceae* across disparate samples).

### Neither eggs nor pupae are likely acting as bottlenecks in microbiota maintenance

**Fig 6:**
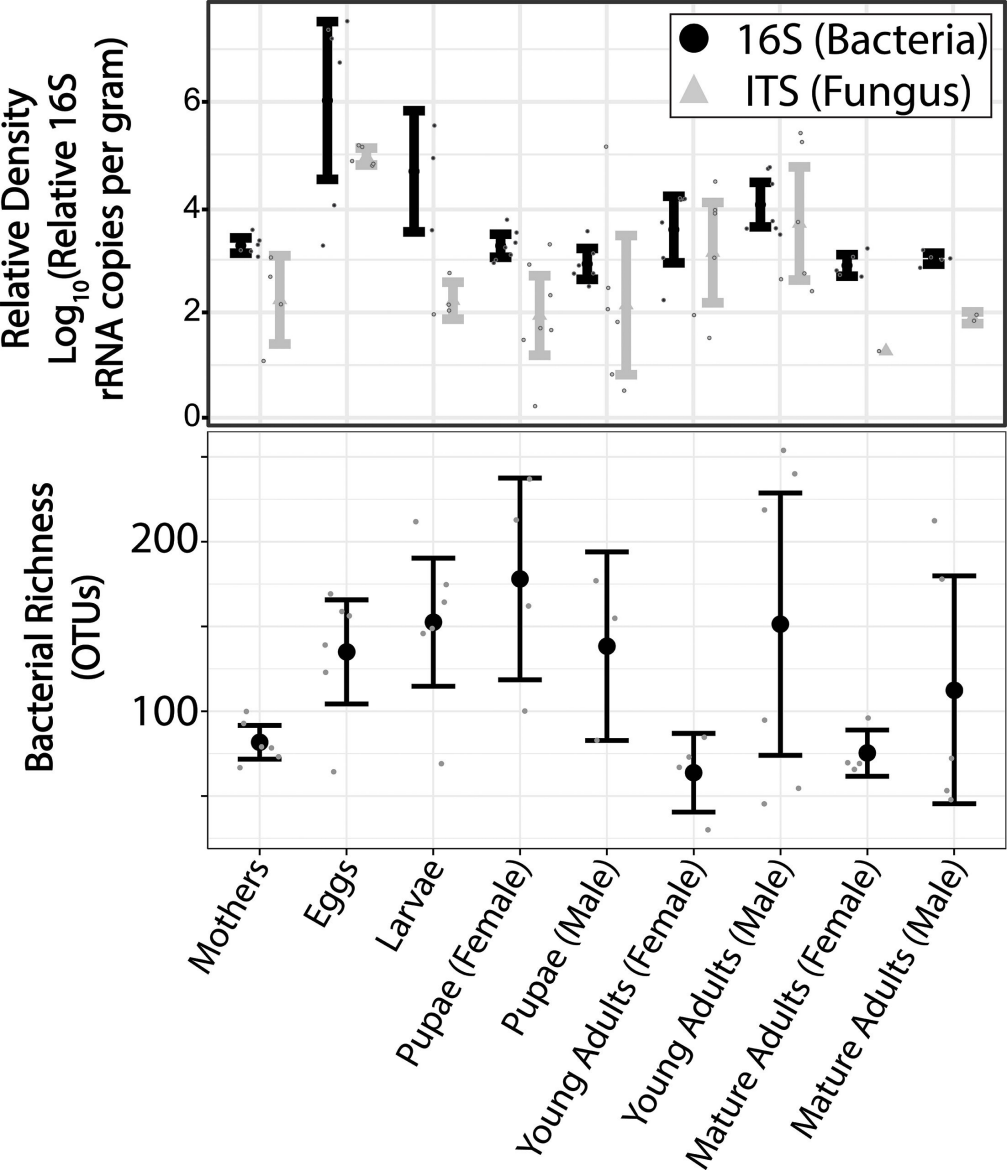
Effect of life stage on microbial density and bacterial richness. (Top) Relative density of 16S (bacterial) and ITS (fungal) amplicons. Life stage has a significant effect on both bacterial and fungal density (*P* < 0.001) and bacterial richness (*P* < 0.0396). Values represent relative DNA quantities, normalized, and divided by the mass of the sample they originated from. Black represents bacteria and gray represents fungus. Large black circles represent mean bacterial density, large gray triangles represent mean fungal density, lines represent 95% confidence intervals, and smaller circles represent individual samples. (Bottom) Bacterial richness. Large points represent means, lines represent 95% confidence intervals, and smaller points represent individual samples.

We sought to test the hypotheses that eggs and pupal stages may impose bottlenecks on microbial communities because they may lack organ systems where microbes typically reside and rarely consume excess resources for microbial consumption. To determine whether there were any bottlenecks in overall microbial populations across development, we quantified the density of bacterial and fungal amplicons in each sample using qPCR. We find that the host life stage has a significant effect on both bacterial and fungal density (one-way ANOVA; Bacteria: F = 9.4694, *P* < 0.001, Fungus: F = 4.4722, *P* < 0.001, Fig. 6, top). However, contrary to our expectation, we found that eggs host a significantly *higher* density of bacteria than any other life stage (TukeyHSD; *P* < 0.001) and a significantly *higher* density of fungus than mothers, larvae, and male and female pupae (TukeyHSD; *P* < 0.02). When eggs are removed from the analysis, density remains significantly affected by life stage in bacteria (TukeyHSD; F = 6.5049, *P* < 0.001) but not fungus (TukeyHSD; F = 1.7287, *P* = 0.1414). Differences between the density of bacteria in pupae and the remaining life stages varied. For example, pupae consistently contained bacterial communities significantly less dense than those of larvae (TukeyHSD; *P* < 0.005) but similar to those of mothers and mature adults (TukeyHSD; *P* > 0.89). Data Set S2 contains pairwise statistics for all qPCR results. This includes comparisons to environmental samples (Fig. S8) which were measured but not discussed because they are unrelated to our focal hypotheses.

However, it is conceivable that only a select portion of the microbial communities may be maintained throughout the egg and pupal stages. This, too, could represent a possible bottleneck to constrain the maintenance of symbionts. In support of this hypothesis, we find that life stage does have a significant effect on observed bacterial richness (one-way ANOVA; F = 2.3389, *P* = 0.0396, Fig. 6, bottom). To test for the existence of specific stage-to-stage changes in bacterial richness, we then compared the richness of each stage to the next and identified two significant instances of changes in richness: the microbiome of eggs maintain a significantly higher bacterial richness than the mothers that oviposited them (Welch’s *t*-test; t = −3.2359, *P* = 0.01761) and female pupae maintain a bacterial richness higher than the female young adults they will eclose into (Welch’s *t*-test; t = 3.5074, *P* = 0.02582). Comparisons between all sample types, including environmental samples, are shown in Supplemental Fig. S9 and metadata are available in our Data Set S2. Generally, these results reject the notion that eggs and pupae represent obvious bottlenecks to microbial maintenance yet provide modest evidence of density and diversity bottlenecks throughout the rest of the life cycle.

### Pedestals and pupal chambers contain microbial communities distinct from those of the egg and pupa

Finally, we sought to determine whether host-constructed pedestals and pupal chambers may serve as a reservoir for symbionts to bypass or complement microbiome transmission during the egg and pupal stages. To do so, we first compared the richness of bacterial OTUs in both host-constructed features to that of their associated beetle life stage but found no significant difference between each pair (Welch’s t-test; egg-pedestal: t = −2.192, *P* = 0.05912, female pupa—female pupal chamber: t = −.064389, *P* = 0.5542, male pupa—male pupal chamber: t = −2.6564, *P* = 0.1096). These results suggest that neither environmental feature harbors a greater diversity relative to its associated life stage. However, this does not exclude the possibility that pedestals and/or pupal chambers may serve as refugia for a select subset of microbial taxa distinct from those maintained within the host. Therefore, we addressed compositional differences using a pairwise PERMANOVA to test whether the communities maintained within the host (eggs and pupae) are distinct from those present externally (within the pedestals and pupal chambers). Comparing Jaccard similarity, we found that the bacterial community composition in the pedestal was indeed significantly different from that of the egg (Pseudo-F = 1.67, R2 = 0.12256, *P* = 0.048) and that, similarly, the bacterial community within pupal chambers of males differed significantly from that of male pupae (Pseudo-F = 3.22, R^2^ = 0.30474, *P* = 0.028). Intriguingly, a corresponding effect was not observed between female pupae and their pupal chamber, which harbor bacterial communities with similar compositions (Pseudo-F = 1.14, R^2^ = 0.1315, *P* = 0.344).

## DISCUSSION

Multicellular organisms commonly rely on communities of symbiotic microorganisms to support their development, supplement their nutrition, and defend against pathogens, among other functions. Yet, many hosts progress through distinct life stages across which the interactions between host and symbiont may diverge greatly. For example, *Rhizobium* and mycorrhizal fungi can increase germination success in legume seeds while increasing both above and belowground biomass as well as reproductive success during later life stages (35, 36). Conversely, humans rely on beneficial bacteria such as *Bifidobacterium* to establish proper immunity in infancy, yet during later life stages these same taxa become rare and less essential (37). Such life stage dependency in host-symbiont interactions is likely common yet little is known about the mechanisms hosts may have evolved to establish and maintain stage-specific relationships across complex life cycles. Here, we used the dung beetle *Onthophagus taurus* as a model organism to investigate the nature of microbiome dynamics and modes of transmission across a complex host life cycle and to begin identifying the bacterial taxa underlying previously observed symbiont benefits to host development and ecology (32–34, 38).

### Bacterial composition diverges consistently throughout host development, but less frequently between sexes

The holometabolous life cycle is often composed of stages whose properties may heavily influence microbiota composition such as host diet, physiology, and environment (18). We find that the host life stage significantly influences microbiota composition, with some of the most dramatic differences observed between developing stages (larvae and pupae) and mature adult stages (mothers and mature adult males and females). This disparity matches striking differences in ecology and behavior across these stages, whereas larvae and pupae develop underground and in complete isolation from conspecifics, with larvae consuming a fibrous diet, mature adults are highly mobile aboveground, aggregate on dung pads, and consume a liquid diet. More generally, these results parallel findings from previous studies in both other dung beetle species (*Euoniticellus intermedius* and *E. triangulatus* [39]; *Copris incertus* [40]), as well as other holometabolous insects (bees: [41, 42]; butterflies: [43]; flies: [44]; wasps: [45]) that demonstrate substantial changes in host-associated microbiota across holometabolous life stages. Importantly, even though our results document consistent shifts in microbial communities as a function of the host stage, we observed that the bacterial communities of mature adult females and males ultimately returned to that of mothers, that is, that of the individuals that had originally initiated the generation under study. This finding suggests a cyclical nature in the succession of *O. taurus* microbiota across the host life cycle, and more generally suggests that *Onthophagus* beetles maintain a complex, dynamic, yet tractable microbiome in the face of changing host factors and environmental circumstances.

Previous work suggested that host sex may also heavily influence microbiome assembly. Specifically, it has been suggested that adult males may lose larval symbionts following pupation because males—in contrast to females—may not be under selection to pass such symbionts on to their offspring (4). Work on *Euoniticellus intermedius* dung beetles partially supports this hypothesis, documenting that adult males harbored a microbiota more diverged compared to the larval microbiota than that of adult females (39). In the present study, however, mature adult *O. taurus* males and females were found to maintain overall similar microbial compositions and dispersions. Why ecologically similar taxa such as *E. intermedius* and *O. taurus* (which are sympatric in parts of their distribution [46]) may nevertheless differ in the degree of sex bias in adult microbiota, is presently unclear and may result from differences in symbiont-provided benefits, the phylogenetic history and/or behavioral ecology of each host taxon, and/or the relative contributions of vertical and horizontal means of microbiome acquisition, suggesting interesting opportunities for future work.

### Egg and pupal stages are unlikely to bottleneck symbionts throughout development

We also sought to assess the hypothesis that egg and pupal stages may represent environments less conducive to the maintenance of microbial communities, for instance, due to the absence of well-differentiated organ systems to house symbionts. While our study uncovered evidence of fluctuations in microbial density across development, we found that *O. taurus* eggs and pupae could maintain a density comparable to and, in the case of eggs, far exceeding those of other life stages. This is in contrast to earlier studies which suggest that insects are typically devoid of free-living microbes during the egg (47–50) or pupal stages (4, 11). However, our results support previous dung beetle sequencing efforts which demonstrate the existence of distinct microbial communities retained within both egg and pupal stages and raise questions regarding the mechanisms that facilitate such maintenance. For example, recent work on *Copris incertus* and *Catharsius molossus* dung beetles revealed that both harbor microbial communities within their eggs (40, 51), with *Copris* also containing a microbial community within the pupal stage. While many other insects also appear to maintain intracellular microbes within the egg (49, 52) and pupal (25, 42, 44) stages, little is known about the mechanisms allowing non-intracellular symbionts to persist during those stages. For example, *Wolbachia*, a common endosymbiont of insects, manipulates host cellular machinery to translocate into the egg and guarantee maintenance (e.g., *via* actin and yolk uptake mechanisms (41, 53, 54). Similarly, *Blochmannia floridanus*, an endosymbiont of carpenter ants, appears to invade midgut tissues, populating bacteriocytes as well as other cells (55). Some research suggests that gut symbionts that survive the mechanical and immunological suppression by the host during metamorphosis may survive within the midgut of the pupae, such as with *Galleria mellonella* and their symbiotic *Enterococcus mundtii* (25). Beyond simply permitting microbes within pupae, some taxa, such as *G. mellonella* (25) and *Anthophora bomboides* (42), may, in fact, rely on this internal storage to prevent pathogenic infection during pupation and diapause, further emphasizing the importance of host and microbial traits in facilitating microbiome maintenance and function. Precise localization of microbial cells within *Onthophagus* eggs and pupa, as well as research into key symbiont traits, will be needed to begin identifying corresponding mechanisms operating during dung beetle development. In addition, more studies of insect microbiomes should include these developmental stages to deepen our understanding of the prevalence of these dynamics across insect phylogeny.

### Beetle-constructed environments provide additional avenues for symbiont maintenance

We found that the bacterial communities contained within two conspicuous host environments—the pedestal, produced by the mother prior to laying the egg, and the pupal chamber, produced by the larva prior to pupation—were distinct from those found within the egg and pupal stages, respectively. This suggests that *O. taurus* beetles may maintain a subset of their microbiota external to the host to bypass egg and pupal development. This conclusion is further supported by several previous studies that documented, across multiple species and contexts, that dung beetles experience decreased developmental outcomes and reproductive success when deprived of their pedestal (32, 34, 38), which can be rescued if larvae are inoculated with pedestal derived, cultured microbiota. Together, this body of evidence suggests that the pedestal community likely contains key symbionts absent from the egg itself, yet critical to normative *Onthophagus* development.

Whether symbionts stored within the pupal chamber are similarly important is less certain. Here it is worth noting that the microbiome of young adult females differentiates measurably from the pupal microbiome yet does so without experiencing a novel source of microbes other than perhaps by taking up microbes from the pupal chamber. Likewise, there is also a corresponding lack of data on the importance of the microbiome in adults beyond morphological metrics (such as body size [32, 33, 38, 56]) which are primarily shaped by larval physiology. One notable exception is the work by Parker et al. (34) which, among others, also assessed adult survival in *Onthophagus vacca* and *O. medius*, and documented a significant reduction thereof in microbiome-deficient or mismatched individuals (34). Future studies are needed to investigate the possible functional significance of microbiota in pupal and adult stages.

### Microbes available in the maternal diet have a strong effect on egg and pedestal microbiomes

The presence of a microbial community within eggs, and that community’s distinction from that of the pedestal, is likely important to the ecology of the beetle and the maintenance of microbes across generations. Yet, both of these communities are dominated by bacteria also present in the maternal diet and contain only a modest relative abundance of host-associated microbiota. The similarity between the pedestal and maternal diet microbiomes is consistent with the notion that the pedestal represents a maternally derived fecal pellet (21) yet stands in contradiction to the finding that the maintenance of this community benefits larval development in a way that purely environmental bacteria do not (32). One possible key difference causing this developmental effect may be the presence of host-associated microbes in the pedestal, such as *Dysgonomonas* and *Brucella*, which are absent from the diet. Though rare in the pedestal, these microbes increase tremendously in abundance across other stages and may have effects disproportionate to their original abundance.

The observation that the internal egg community consists of primarily environmental taxa raises further questions. Yet, such a correspondence between egg and environmental microbiota has also been observed in *Copris incertus* and *C. molossus,* including *Acinetobacter*, *Sphingobacterium*, and *Nocardiodes*, suggesting that their presence within dung beetle eggs may be common (40, 51). Whether these microbes colonize the egg prior to it being laid or after is yet to be determined; however, *O. taurus* have been shown to host communities of nematodes on and within their internal genitalia, which suggests that the reproductive systems of these beetles may not be entirely sterile. Furthermore, ovary microbiomes have been studied in other systems, such as the cereal weevil (57), yet these communities are housed within bacteriocytes and are much simpler than those observed here.

### Core microbes of potential host benefit

This study uncovered a great diversity of bacterial taxa associated with parts, or all, of the *O. taurus* life cycle. Among them lies a subset of taxa which, given their relative abundance during key host stages and/or presumed metabolic ability, represent promising candidates for influencing host fitness. For example, *Dysgonomonas* were found throughout most of the host life cycle and reached their highest abundance during the larval stage. Recall that larval *O. taurus* undergoes rapid mass gain by consuming the fibrous, cellulose-rich dung within the brood ball. Earlier work suggests that *Dysgonomonas* can break down cellulose (58, 59) and has been associated with other cellulose-feeding insects (such as termites [60], cockroaches [61], carpenter bees [62], and dung beetles [40, 63]). Moreover, *Dysgonomonas* were also found abundantly in the pupal chamber suggesting that they may have the potential to survive both within the beetle and the beetle’s constructed environment. By extension, this would support the hypothesis that *Onthophagus* beetles may actively enrich the external environments surrounding them during larval development with metabolically active symbionts, constructing an external rumen in the process (64). Finally, it is important to highlight that *Dysgonomonas* has been observed in dung beetles across four different studies, including *Copris incertus* (40), *Phanaeus vindex* and *P. difformis* (65), *Euoniticellus intermedius* and *E. triangulatus* (39), *C. molossus* (51), *Onthophagus binodis* (66), *O. australis*, *O. hecate*, *E. fulvus,* and three populations of *O. taurus*, spanning three continents (63). Our data, in conjunction with past results, thus suggest that *Dysgonomonas* may constitute an important member of the *Onthophagus* core microbiome which increases in abundance during the larval stage and facilitates the stage-specific breakdown of the larval brood ball.

*Acinetobacter* and *Brucella* also represent putative core microbes that may influence host development and fitness. *Acinetobacter* has been described primarily in the context of floral and bee microbiomes (67–69), where it has evolved the ability to digest floral resource and seems to also play a role in degrading plant defenses within the weevil gut (70). In dung beetles, *Acinetobacter* typically co-occurs with *Dysgonomonas* (39, 40, 51, 63, 65, 66) and has also been documented in the dwelling dung beetle *Aphodius fossor* (71). By contrast, *Brucella* is almost entirely viewed as a pathogen in mammals (72). Yet in *O. taurus*, *Acinetobacter* and *Brucella* represent the most sequenced microbes across several stages. Further experimental work will be required to determine whether and how these genera influence host development or fitness. Further surveys are also needed to determine how common these taxa are across dung beetle diversity.

### Conclusions

*Onthophagus taurus* represents a useful model for understanding how host organisms may maintain as well as modulate their microbiota across development. Here we document that *O. taurus* plays host to a diverse microbiota that undergo drastic community shifts throughout host development, influenced by host life stage, environmental microbiota, and, to a lesser degree, host sex. Contrary to our predictions, we found that egg and pupal stages do not constrain the maintenance of key microbes, while host-constructed environments (pedestal, pupal chamber) may serve as complementary microbial refugia for select taxa. Lastly, despite the fluctuations of microbiota throughout development, we identify a small community of putative core microbiota, several of which are likely to play key roles in shaping host development and fitness.

## MATERIALS AND METHODS

### Sample collection and preparation

Wild-caught *Onthophagus taurus* were collected near Chapel Hill, North Carolina, USA, maintained in the laboratory, and bred to produce an F1 population of adults following established methods (73). Beetles were fed homogenized dung collected from Marble Hill Farm in Bloomington, IN throughout the experiment. F1 progeny were maintained in a colony for 3 weeks to reach sexual maturity and ensure females became inseminated. Afterward, select females were placed into individual breeding containers to produce brood balls for sample collection. These females, henceforth referred to as mothers, were allowed two 5-day increments for brood ball production. Mothers were moved to a fresh breeding container after the first increment. In total, 26 mothers produced an average of 6.4 brood balls (range: 0–18). Brood balls produced by each female were collected in separate containers to establish seven family lines for subsequent sample collection (family count is lower than starting female count because we did not use offspring of mothers that died prior to collection or families with fewer than eight total brood balls).

Mothers were frozen at −80°C after brood ball production and stored at −80°C for later processing. Brood balls were opened and offspring were transferred into standardized artificial brood balls (ABBs) contained within 12-well plates filled with dung from grass-fed cows (a standard method detailed in reference 74). At the time of transfer, offspring were either in the egg stage and thus transferred alongside their pedestals, or as very early first instar larvae, which had already consumed their pedestals. Plates were placed within an incubator at 24°C with a 16:8 h light:dark cycle to allow offspring to continue their development. In addition, one brood ball per family was harvested to obtain an egg and a pedestal sample. Using this approach, the following 12 samples were collected: mother, egg, pedestal, larva (5 days after reaching their final [=3rd] instar), male and female pupa (4 days after larva entered the prepupa stage), pupal chambers produced by larvae prior to pupation, and both male and female adults immediately after eclosion (young adults) and after 3 weeks of living, feeding, and interacting within a colony (mature adults), respectively. Sampling was based on maternal lineage; however, maternal survival to sampling was low (*n* = 7) and sampling of offspring was incomplete in some of these matrilineages. Analyses related to family were therefore dropped. Samples were also collected from the homogenized dung used in the breeding containers and the 12-well plates, prior to utilization for the experiment, to capture environmental microbiota.

To prepare for whole-body DNA extraction, all beetle samples were surface sterilized with a 1% bleach solution and a 70% ethanol solution followed by a rinse in sterile PBS. In pilot data, sterilization of eggs with this method is adequate to eliminate living microbes from the surface of the eggs (i.e., as determined through the absence of any colony-forming units resulting from rolling an egg on the surface of an agar plate). Furthermore, sterilization with these methods does not eliminate an internal community of microbes (i.e., as assessed by the ample presence of colony-forming units observed from a homogenate of a sterilized egg on an agar plate). Environmental samples, including the pedestal, pupal chamber, and dung were not sterilized prior to extraction. All samples were frozen in liquid nitrogen and ground with a mortar and pestle followed by DNA extraction using a Qiagen DNeasy PowerSoil Pro Kit in a randomized order. A negative control was also run, absent of any sample, to identify any potential contamination.

### Amplicon sequencing and quality control

To determine the bacterial community composition within samples, we amplified the V4 region of the 16S rRNA gene (515F-806R) in all DNA extracts using protocols modified from the Earth Microbiome Project’s 16S Illumina Amplicon Protocol (515F: AATGATACGGCGACCACCGAG ACGTACGTACG GT GTGCCAGCMGCCGCGGTAA, 806R: CAAGCAGAAGACGGCATACGAGAT XXXXXXXXXXXX AGTCAGTCAG CC GGACTACHVGGGTWTCTAAT, “X” represent 12 base pair barcode sequences which differ across samples) (75). We also attempted to amplify ITS DNA for analyzing fungal community compositions but amplifications, using ITS1f (AATGATACGGCGACCACCGAGATCTACA CGG CTTGGTCATTTAGAGGAAGTAA) and ITS2 (CAAGCAGAAGACGGCATACGAGAT XXXXXXXXXXXX AGTCAGTCAG AT GCTGCGTTCTTCATCGATGC) primers (76) modified with Illumina barcodes and adaptor sequences, were unsuccessful resulting from an overamplification of primer-dimers. It is important to note that the primers used for this amplification are distinct from those used for the qPCR. PCRs were conducted in a Mastercycler Gradient Thermocycler using an HF Phusion polymerase mix. The negative controls amplified no DNA and were not processed any further. Prior to sequencing, non-target amplifications were removed using QIAquick Gel Extraction kits, and libraries were pooled, based on fluorescence using the Quant-iT PicoGreen dsDNA Assay Kit. The pooled libraries were cleaned using the QIAquick PCR Purification kit and sequenced across two MiSeq 500 v2 Nano flow cells at Indiana University’s Center for Genomics and Bioinformatics. The resulting amplicon sequences were demultiplexed, with adaptor sequences removed, and processed through *Mothur* (77). Amplicon sequences were trimmed to 275 base pairs and ambiguous bases were removed. Unique sequences were aligned to the SILVA v138.1 16S rRNA reference database (78) and trimmed to eliminate insertions past the terminal ends of the alignments. Sequences identified as chimeras, Eukaryota, Archaea, mitochondria, and chloroplasts were removed. Finally, the remaining amplicons were clustered at 97%, taxonomically, identified using the consensus taxonomy for that cluster against the SILVA database, and rarefied to 1,013 reads per sample, removing those samples with less (Rarefaction curve: Fig. S1). The occurrence of each cluster within each sample was exported to R for statistical analysis. A single male pupa sample was removed from the analysis (B_MP) on account of an abundance of environmentally associated taxa greatly exceeding that of other pupae, suggesting infection. Sample sizes used for final analyses can be found in Table S1. In addition, the most abundant *Brucellaceae* OTU (operational taxonomic unit; OTU002) was identified using NCBI’s BLAST (79), identified as *Brucella*, and referenced as such in analysis, figures, and discussion.

### qPCR for relative density of microbes

To quantify the total abundance of microbiota relative to other samples, the relative abundance of 16S (bacterial DNA; 515F-806R) and ITS (fungal DNA; ITS1f-ITS2R) DNA copies were measured across samples using qRT-PCR. To do so, DNA extracts from each sample were amplified with Quantbio’s PerfeCTa SYBR Green, Low Rox, in triplicate in a QuantStudio 6 Flex machine. 16S cycle conditions: 95°C for 10 minutes, followed by 45 cycles of 95°C for 15 s, 53.2°C for 30 s, and 72°C for 15 s. The melt curve was measured with a temperature change of 1.6 C/s starting at 95°C for 15 s to 60°C for 1 min to 95°C for 15 s. ITS cycle conditions: 95°C for 10 minutes, followed by 45 cycles of 95°C for 15 s, 55.5°C for 30 s, and 72°C for 15 s. The melt curve was measured with a temperature change of 1.6°C/s starting at 95°C for 15 s to 50°C for 1 min to 95°C for 15 s. Standards across both utilized one of the pupal chamber samples at five different dilutions (1, 0.2, 0.04, 0.008, and 0.002) the results of which were used to calculate primer efficiency. After qPCR and data quality control, relative DNA quantities were calculated by multiplying the primer’s efficiency (e) by the power of the negative CT value (DNA = e^−CT^). The efficiencies were calculated at 110% for 16S and 89% for ITS. Relative density was then calculated by dividing relative DNA quantity by sample mass and log10 transforming it log_10_(DNA/mg). Data for qPCR efficiencies are provided in Data Set S2. CT values, sample masses, and calculated densities are available in Data Set S2.

### Statistical analysis

All statistical analysis was conducted using R version 4.2.2. Relative abundance of taxa, or groups of taxa, represent the proportion of rarefied reads associated with that taxon/group. Factors influencing the density of microbes, bacterial richness, and the relative abundance of environmentally associated bacteria were determined using one-way ANOVAs. Significant differences between sample types were determined using either Tukey’s HSD, for all pairwise comparisons, or Welch’s *t*-test, for data pertaining to stages that occur sequentially throughout development, including the relative abundance of dominant taxa and relative density of microbes across life stages. PERMANOVAs were used to determine variables affecting beta diversity between bacterial communities within samples as well as to determine which sample types contain significantly different communities. Differences in variance between sample types were determined using BetaDisper, from the *vegan* package (80). Finally, an indicator species analysis was conducted, using the *indicspecies* package (81), to determine correlations between bacterial taxa and sample types.

## Data Availability

All 16S sequences are accessible in the NCBI Sequence Read Archive (SRA) under the BioProject number PRJNA1029197. Additional data, including pre- and post-rarified OTU tables, OTU taxa classifications, qPCR efficiency data, and the metadata for the density and richness calculations (sample life stages, richness, 16S and ITS CT values, and masses), are available in Data Set S2.

## SUPPLEMENTAL MATERIAL

Data Set S1 aem.01278-24-s0001.xlsx Complete statistical results for pairwise comparisons.

Data Set S2 aem.01278-24-s0002.xlsx Metadata for richness and density as well as OTU classifications and site x species matrices.

Supplemental material aem.01278-24-s0003.pdf Figures S1 to S9; Table S1.

ASM does not own the copyrights to Supplemental Material that may be linked to, or accessed through, an article. The authors have granted ASM a non-exclusive, world-wide license to publish the Supplemental Material files. Please contact the corresponding author directly for reuse.
