# Supplementary material for: Microbiome composition and turnover in the face of complex lifecycles and bottlenecks: insights through the study of dung beetles
Source: Appl Environ Microbiol. 2024 Dec 20;91(1):e01278-24. doi: 10.1128/aem.01278-24 (PMC11784073; doi:10.1128/aem.01278-24)
Supplement: Supplemental material — Figures S1 to S9; Table S1. [file aem.01278-24-s0003.pdf]

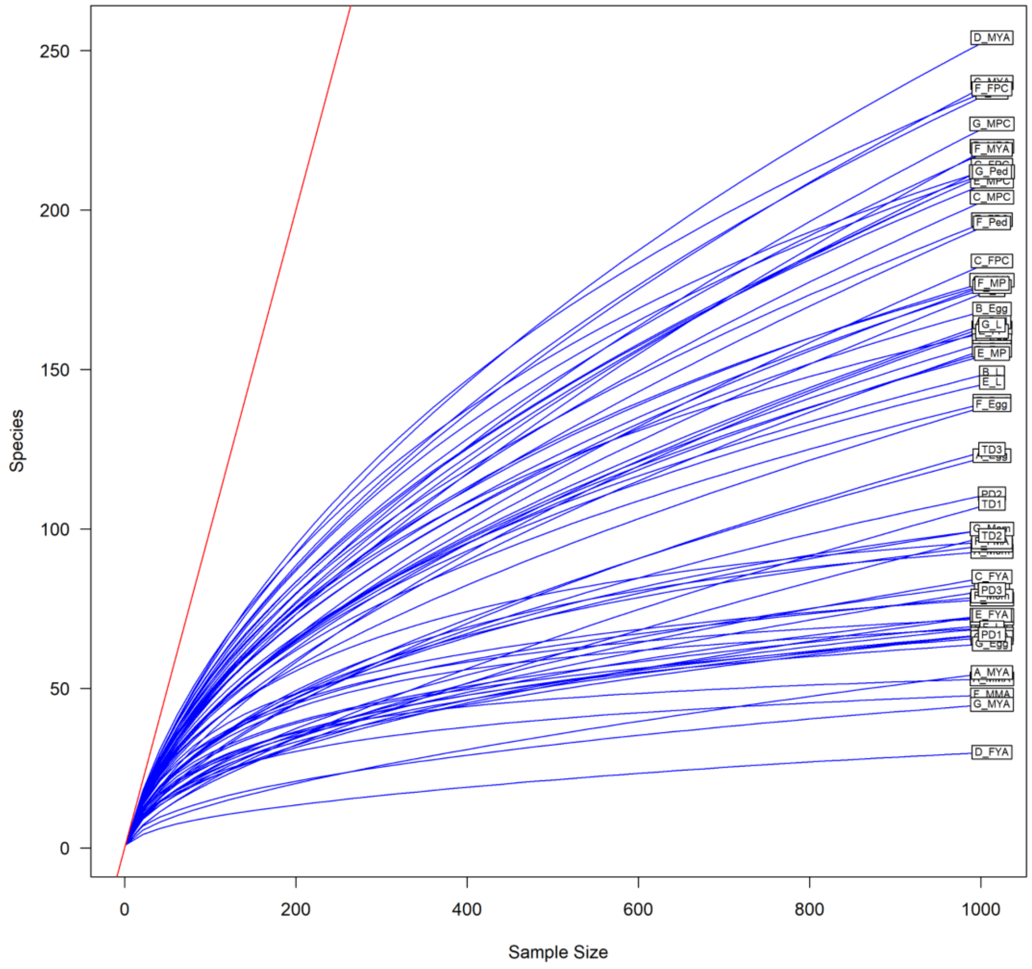

Supplemental figure 1. Rarefaction curves for samples used in bacterial community analysis. Ranges between 0 and 1000 are shown.

Sample type	Data type: Amplicon composition	16S Density	ITS Density
Egg	6	6	4
Female Mature Adult	4	6	5
Female Pupae	4	5	7
Female Pupal Chamber	5	3	4
Female Young Adult	4	7	7
Larvae	6	3	3
Male Mature Adult	5	7	7
Male Pupae	3	2	2
Male Pupal Chamber	4	6	6
Male Young Adult	6	7	6
Mothers	6	5	1
Pedestals	7	5	2
Plate Dung Samples	3	2	3
Breeding Dung Samples	3	0	0

Supplemental table 1. List of samples sizes for bacterial composition and density analyses.

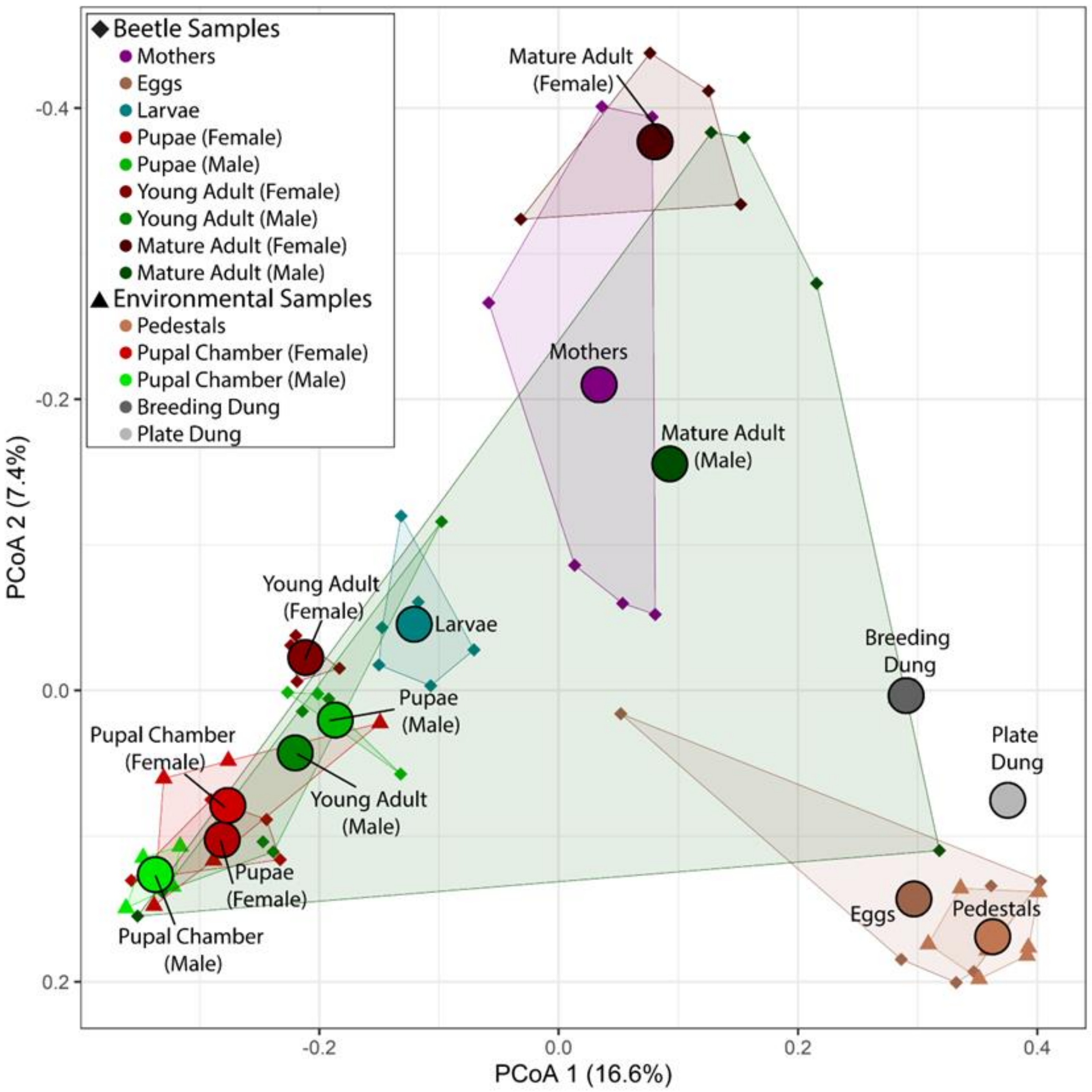

Supplemental figure 2. Jaccard simmilarly of microbial communities hardbored within beetle life stages and environments . Shown are PCoA axes 1 & 2. Sample types are differentiated by colors, diamonds represent beetle samples, triangles represent environmental samples, circles represent each sample type's centroid, and colored lines depict a hull outlining sample type.

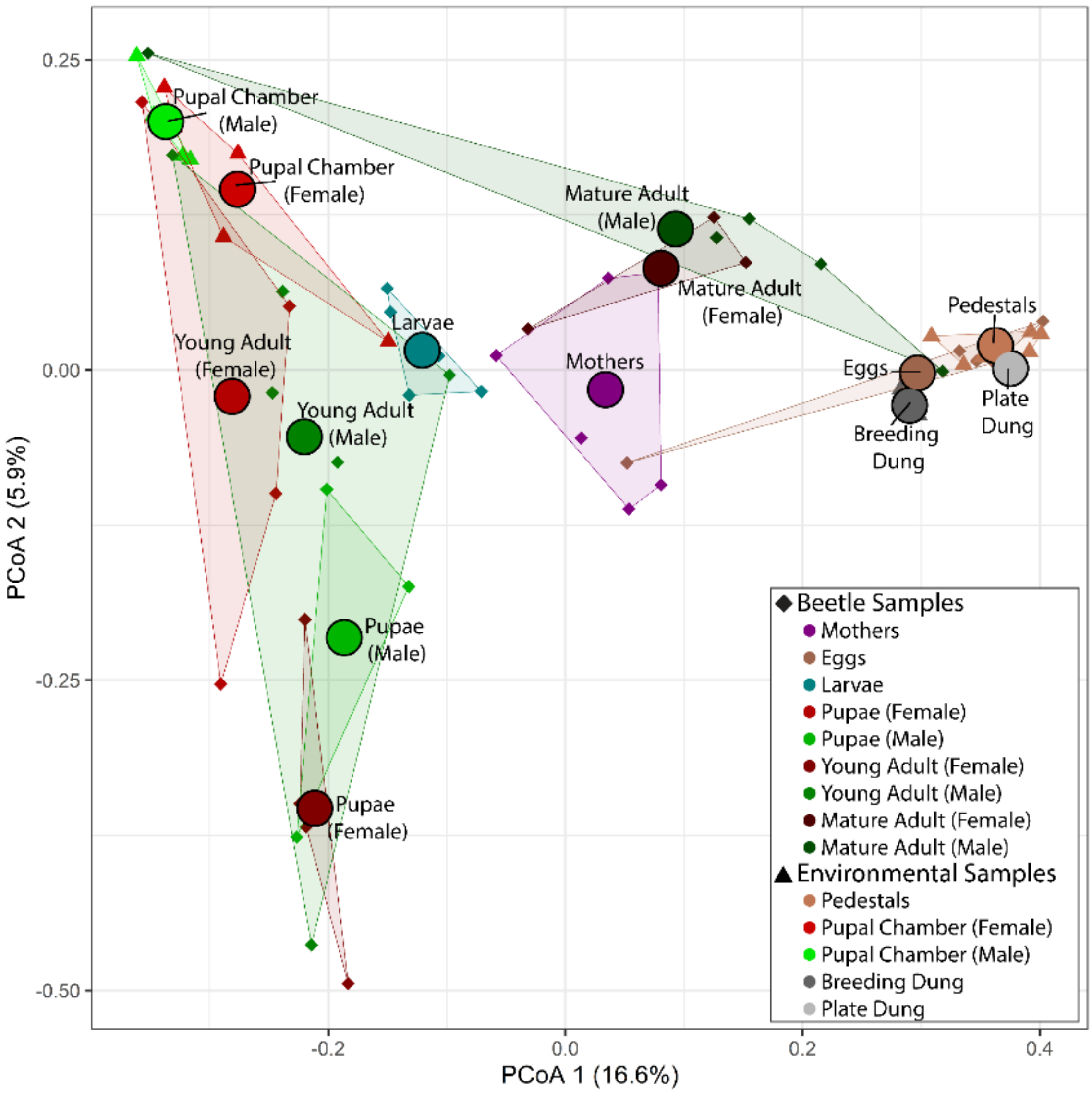

Supplemental figure 3. Jaccard simmilarly of microbial communities hardbored within beetle life stages and environments . Shown are PCoA axes 2 & 3. Sample types are differentiated by colors, diamonds represent beetle samples, triangles represent environmental samples, circles represent each sample type's centroid, and colored lines depict a hull outlining sample types.

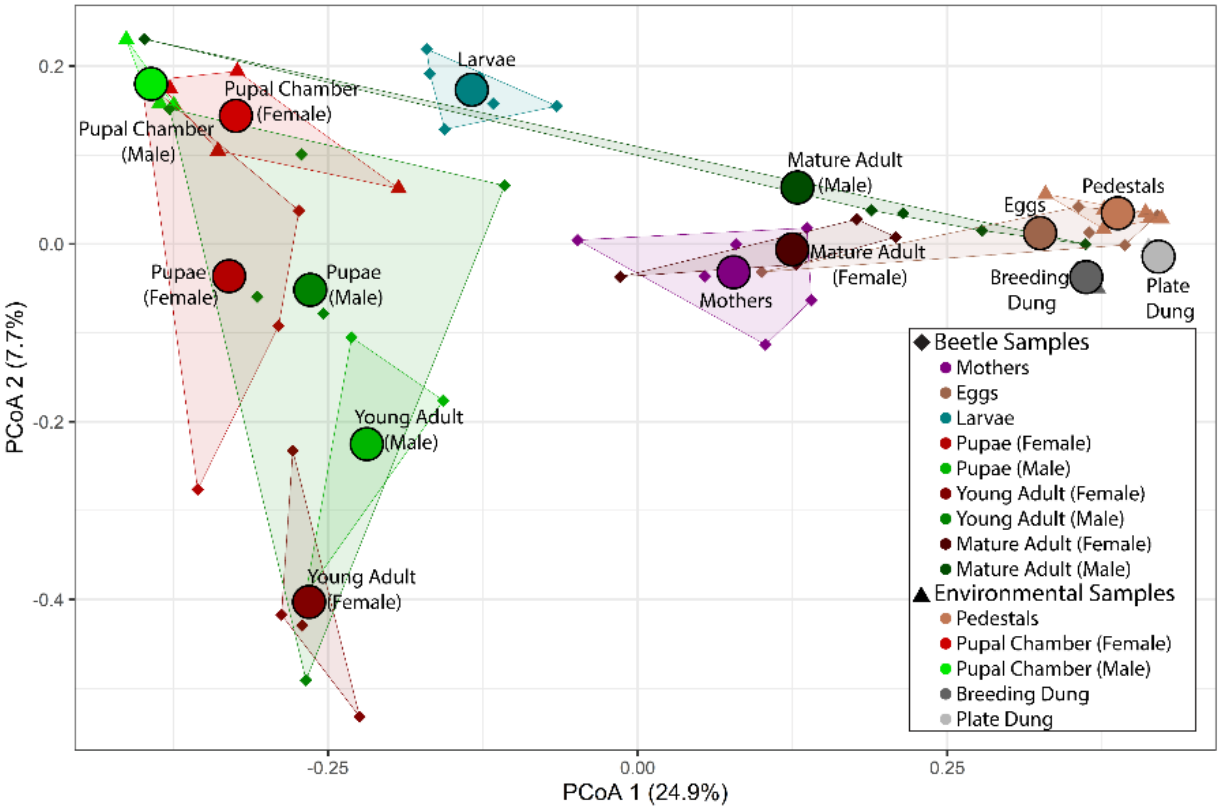

Supplemental figure 4. Bray-Curtis dissimilarity of microbial communities hardbored within beetle life stages and environments . Shown are PCoA axes 2 & 3. Sample types are differentiated by colors, diamonds represent beetle samples, triangles represent environmental samples, circles represent each sample type's centroid, and colored lines depict a hull outlining sample types.

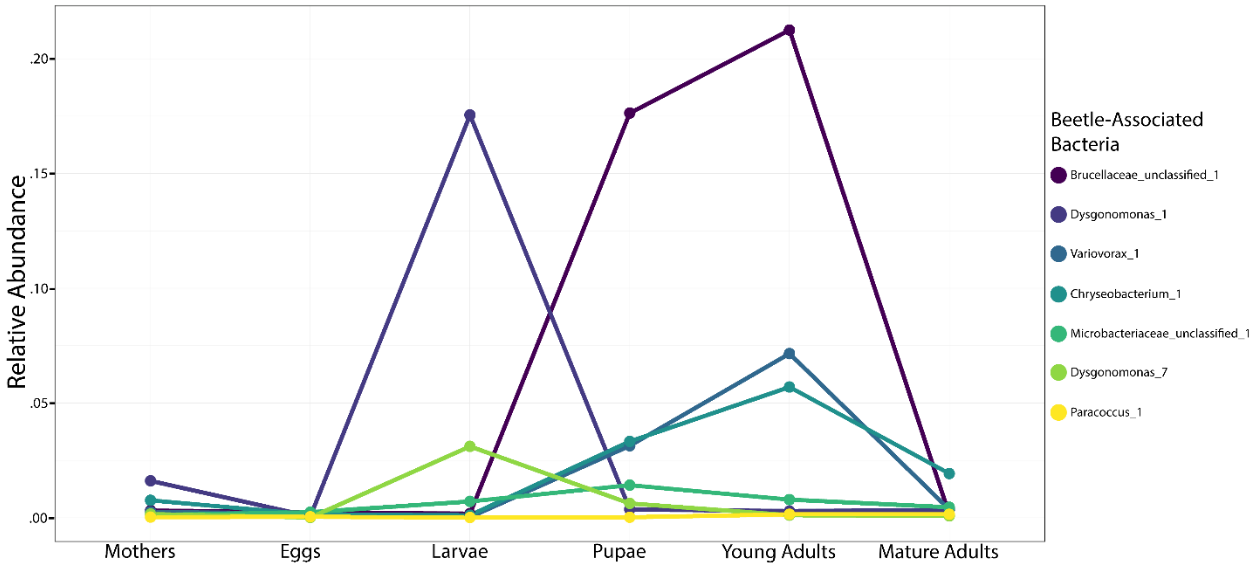

Supplemental figure 5. Putative core beetle-associated microbes. Average relative abundance of bacterial OTUs found across all beetle life stages and not found in any dung sample. Colors correspond with individual OTUs and points are averages across each individual within that life stage.

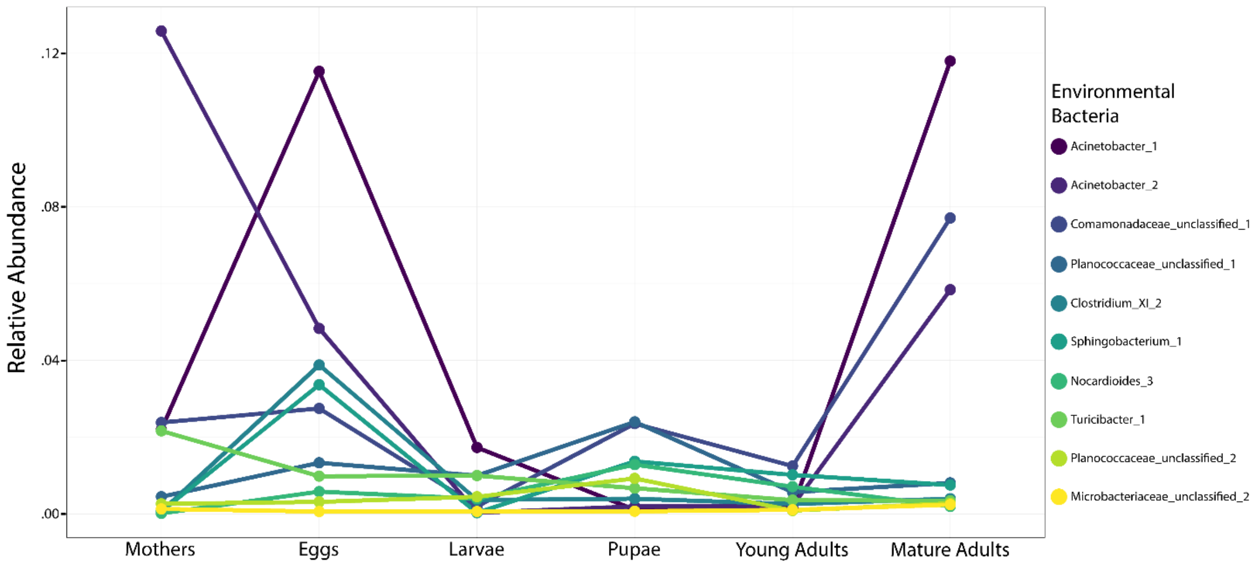

Supplemental figure 6. Putative core environment-associated microbes. Average relative abundance of bacterial OTUs found acorss all beetle life stages and in at least one dung sample. Colors correspond with individual OTUs and points are averages across each individual within that life stage.

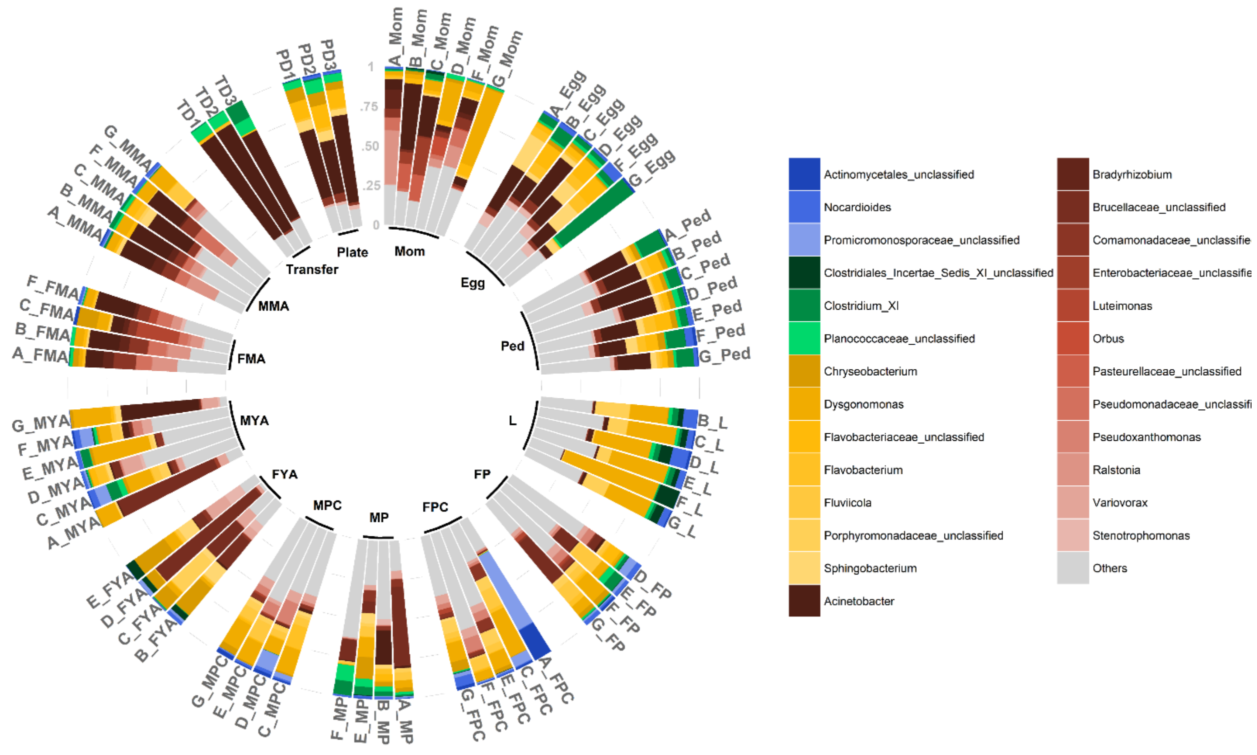

Supplemental figure 7. Relative abundance of bacterial genera across each sample. Samples are grouped based on sample type and shown in order of life stage and associated environment. Phylum is differentiated by color and genus is differentiated by shade. Genera depicted are those that represented at least 10% relative abundance in at least one sample.

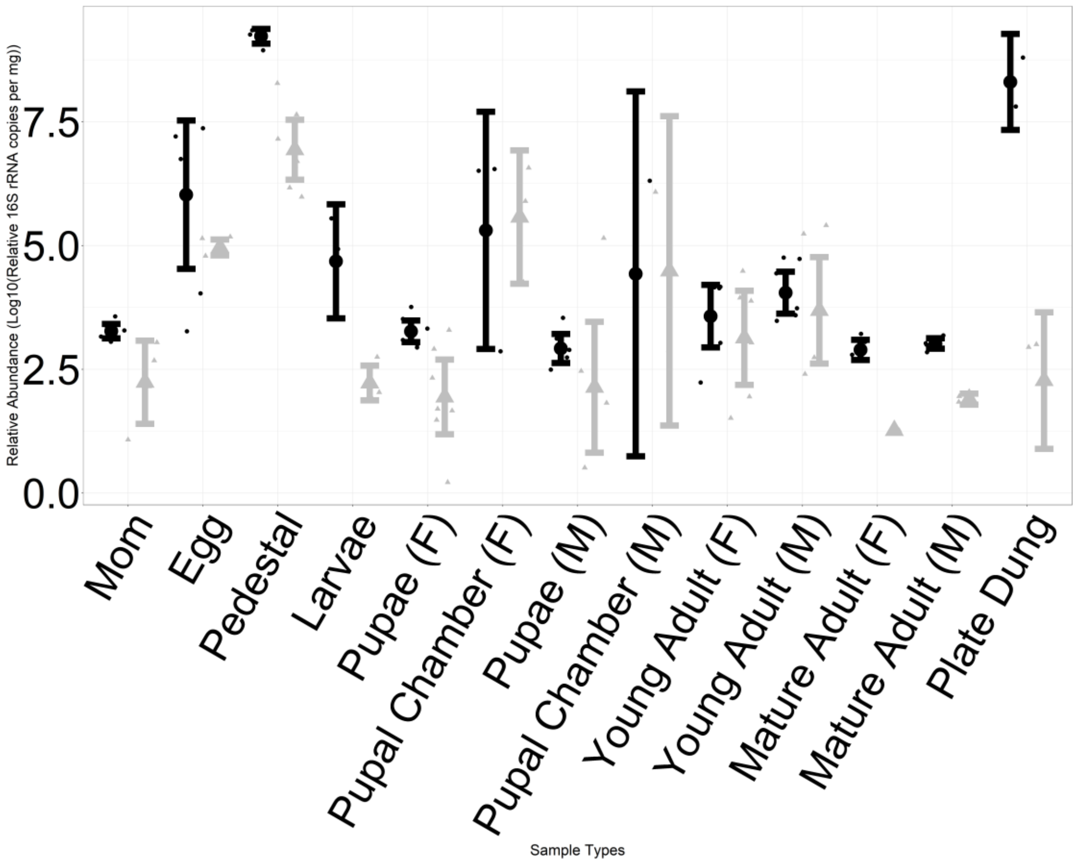

Supplemental figure 8. Effect of sample type on microbial density. Relative density of 16S (bacterial) and ITS (fungal) amplicons. Values represent relative DNA quantities, normalized, and divided by the mass of the sample they originated from. Black represents bacteria and gray represents fungus. Large black circles represent mean bacterial density, large gray triangles represent mean fungal density, lines represent 95% confidence intervals, and smaller circles represent individual samples.

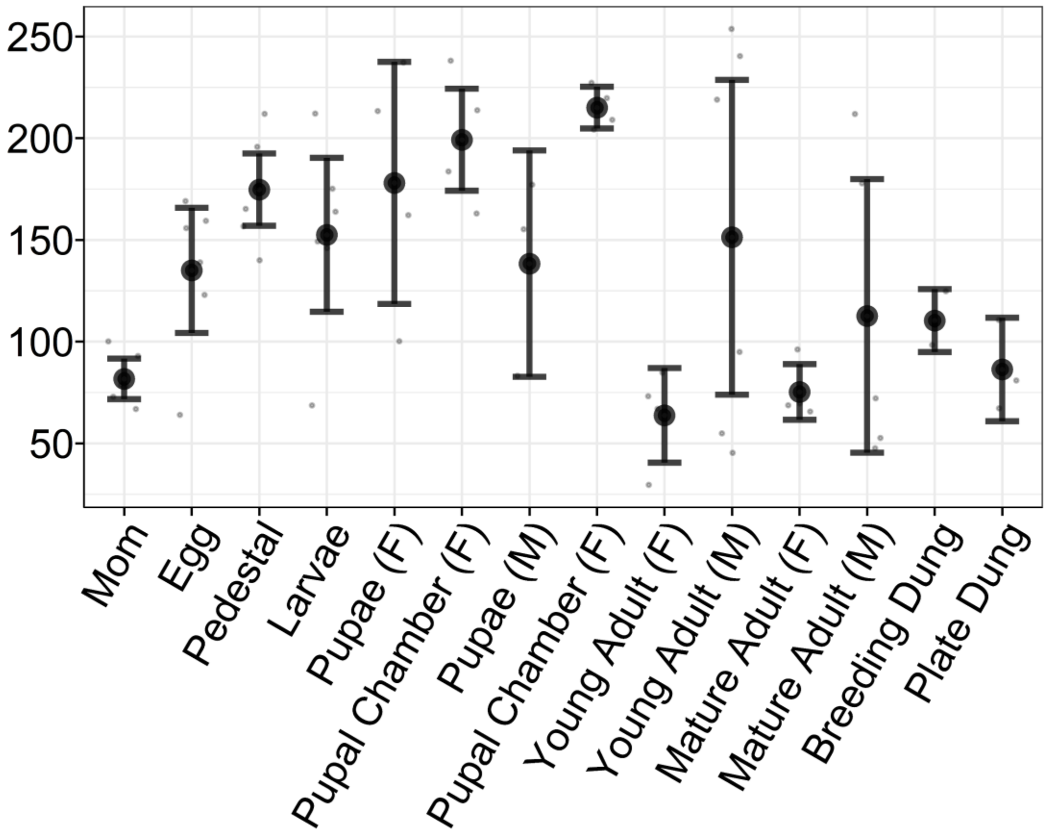

Supplemental figure 9. Effect of sample type on bacterial richness. Large points represent means, lines represent 95% confidence intervals, and smaller points represent individual samples.
